# Monotonous driving induces shifts in spatial attention as a function of handedness

**DOI:** 10.1038/s41598-021-89054-1

**Published:** 2021-05-12

**Authors:** D. Chandrakumar, S. Coussens, H. A. D. Keage, S. Banks, J. Dorrian, T. Loetscher

**Affiliations:** Cognitive Ageing and Impairment Neurosciences Laboratory, Behaviour-Brain-Body Research Centre, School of Psychology, Justice & Society, University of South Australia, GPO Box 2471, Adelaide, SA 5001 Australia

**Keywords:** Human behaviour, Attention

## Abstract

Current evidence suggests that the ability to detect and react to information under lowered alertness conditions might be more impaired on the left than the right side of space. This evidence derives mainly from right-handers being assessed in computer and paper-and-pencil spatial attention tasks. However, there are suggestions that left-handers might show impairments on the opposite (right) side compared to right-handers with lowered alertness, and it is unclear whether the impairments observed in the computer tasks have any real-world implications for activities such as driving. The current study investigated the alertness and spatial attention relationship under simulated monotonous driving in left- and right-handers. Twenty left-handed and 22 right-handed participants (15 males, mean age = 23.6 years, *SD* = 5.0 years) were assessed on a simulated driving task (lasting approximately 60 min) to induce a time-on-task effect. The driving task involved responding to stimuli appearing at six different horizontal locations on the screen, whilst driving in a 50 km/h zone. Decreases in alertness and driving performance were evident with time-on-task in both handedness groups. We found handedness impacts reacting to lateral stimuli differently with time-on-task: right-handers reacted slower to the leftmost stimuli, while left-handers showed the opposite pattern (although not statistically significant) in the second compared to first half of the drive. Our findings support suggestions that handedness modulates the spatial attention and alertness interactions. The interactions were observed in a simulated driving task which calls for further research to understand the safety implications of these interactions for activities such as driving.

## Introduction

Driving safely relies on the ability to sufficiently attend to and navigate the environment^1^. When driving, one must complete multiple tasks simultaneously, such as attending to passing cars, pedestrians, traffic signs and react accordingly, whilst also maintaining to a speed limit and driving within the designated lane. Prolonged driving durations can lead to lapses in attention and consequently increase the risk of experiencing a traffic accident^2–4^. Approximately 10–25% of fatal road accidents worldwide result from attentional failure, where the ability to detect and respond to road-traffic information is impaired^5–8^. In Australia, such attention-related road accidents are estimated to cost the economy approximately 3 billion dollars per year^9^.

Reduced driver alertness is a common reason for attention-related accidents^10^. Alertness is defined as the preparedness to respond to stimuli^11^ and typically decreases whilst engaging in a task for extended durations^12–14^. This decrease in alertness with extended duration is known as the time-on-task effect^15^. Studies investigating simulated driving performance show decreased alertness with 30 min of driving under monotonous conditions. In such instances, the vehicle’s position deviates horizontally from the centre of the lane, and the ability to identify and react to signals decreases with time-on-task^16,17^.

Studies examining driving performance overlayed with a secondary task, which involves detecting information in the horizontal periphery, report a tunnelling of vision with time-on-task^16,17^. These studies reported a reduction in the ability to detect and react to information in the periphery compared to the centre of the visual field with prolonged task exposure. However, it is unclear whether performance decrements in the periphery are uniform with time-on-task under driving conditions, such that both the left and right sides are equally impaired.

Current evidence suggests that the ability to detect and react to information under lowered alertness conditions might be more impaired on the left than the right side of space, when assessed with computer and paper-and-pencil spatial attention tasks^18,19^. A recent meta-analysis revealed attention in healthy adults shifts towards the right with lowered alertness in laboratory-based spatial attention tasks^20^. This rightward shift is commonly demonstrated by slowed responses to left-sided stimuli^21,22^ and the misidentification of left-sided stimuli as being on the right^23^.

The current theoretical model proposed to account for the relationship between spatial attention and alertness involves the bilateral dorsal attentional network (DAN) which is responsible for attention allocation to the contralateral visuospatial field, and the right-lateralised ventral network (VAN), which is involved in detecting unattended stimuli, reorienting attention and alertness. These two networks are in dynamic interactions with each other and changes to alertness levels (VAN) can influence the interactions between these two neural networks and consequently influence attentional allocation (DAN). These interactions result in higher and lower levels of alertness leading to leftward and rightward shifts in attention, respectively^24^.

One study linked changes in EEG alpha power with changes in spatial attention in a time-on-task paradigm in healthy participants^19^. Alpha power provides estimates of cortical activation and deactivation, where alpha-indexed deactivation is associated with an active brain state^25^. Using EEG techniques, the authors showed an increase of pre-target alpha absolute power over the right compared to left hemisphere with time-on-task, and this shift also correlated with a behavioural rightward shift in attention^19^. Lower alpha power in regions contralateral to the attended visuospatial field is apparent during task performance^26^, and previous studies have associated right-lateralised cortical regions with time-on-task^27,28^. This suggests that right hemisphere activation associates with increasing time-on-task, and consequently supports the right hemisphere being implicated in alertness.

Research examining spatial attention and alertness has mostly been confined to right-handers, with left-handers being excluded from study participation^29^. However, the neural structures postulated for the attentional networks involved in mediating the relationship between alertness and spatial attention may differ (to some degree) between left- and right-handers. A fMRI neuroimaging study reported the VAN (which is right-lateralised in right-handers) to be bilateral or left-lateralised in their left-handed sample^30^. However, in contrast to these findings, another study suggests the VAN to be right lateralised in both a left-handed sample and a right-handed sample, whereas the authors found the DAN to be more pronounced in the right hemisphere for left-handers^31^. While there are some discrepancies between the findings in these neuroimaging studies, they suggest that the DAN and VAN networks may differ between left-handers and right-handers. If so, the relationships between alertness (VAN) and spatial orienting (DAN) may differ between left- and right-handers. This was indeed confirmed by the only study exploring handedness influences on alertness and spatial attention. The study found that left- and right-handers show an opposite behavioural spatial attention and alertness relationship pattern. While decreasing alertness led to a rightward shift in the right-handed group, the alertness decrease led to a leftward shift in the left-handed group^32^. Hence, handedness may be an important modulator in the relationship between spatial attention and alertness.

The current study sought to examine the relationship between spatial attention and alertness under simulated driving in left-handers and right-handers using both behavioural and physiological measures of alertness. We expect that physiological and behavioural indices of alertness and driving performance will decrease with time-on-task. We also expect that handedness influences the spatial attention and alertness relationship with time-on-task. More specifically, we hypothesise in right-handers that: (1) there will be slowed response times and more errors on the left side with time-on-task; (2) alpha power over the right hemisphere will decrease with time-on-task; (3) these decreases in physiological alertness will associate with poorer spatial performance on the left than right. For left-handers, we hypothesise the opposite lateral pattern with: (1) slower response times and more errors on the right side with time-on-task; (2) alpha power over the left hemisphere will decrease with time-on-task; and (3) decreases in physiological alertness will associate with poorer spatial performance on the right than left.

## Method

### Participants

A previous study investigating a sample of left-handers and right-handers^32^ found effects of *d* = 0.71 (reaction time) and *d* = 1.29 (error rates) when investigating alertness and spatial attention. The average of these effect sizes was entered into G*Power^33^ for a priori sample size calculations in a repeated-measures, within-between interaction design with a power of .9 and alpha set at .05. This yielded a sample size of 46 participants.

Forty-six healthy participants were recruited with the following inclusion criteria: aged between 18 and 40 years, native English speakers, no hearing or visual impairments that are not corrected for, no history of psychiatric or neurological diagnosis, no current or previous (within 1 year) history of alcohol or substance abuse, no use of recreational drugs within the last three months, have not taken any medication that target the central nervous system (e.g., anticonvulsants, antidepressants) in the last month, no history of unconsciousness greater than 5 min, no known intellectual impairments, and a valid driver’s licence. Four participants were excluded from analysis due to the following issues: inability to perform the task due to falling asleep (i.e., participant was woken up by researcher several times; n = 1), technical difficulties with equipment (n = 2), and a high proportion of errors (i.e., failure to detect and respond to more than 50% of stimuli; n = 1).

The final sample comprised of 42 participants (15 males) aged between 18 and 37 years (mean age 23.6 ± 5 years). Twenty-two participants were right-handed and 20 participants were left-handed, assessed using the FLANDERS handedness survey^34^. Participants in the final sample had an average of 4.6 years of driving experience. Recruitment was via flyers on the University of South Australia’s Magill Campus, social media, and through the University’s online SONA system (Sona Systems Ltd.) which requires potential participants to be invited to participate if deemed eligible. Separate flyers for recruiting only left-handers were distributed via the same method outlined above.

Participants received reimbursement of $15 (AUD) per hour of participation for their involvement in the study. This study was approved by the Human Research Ethics Committee from the University of South Australia.

## Stimuli and apparatus

### Behavioural data acquisition

#### Driving simulator task

**Figure 1:**
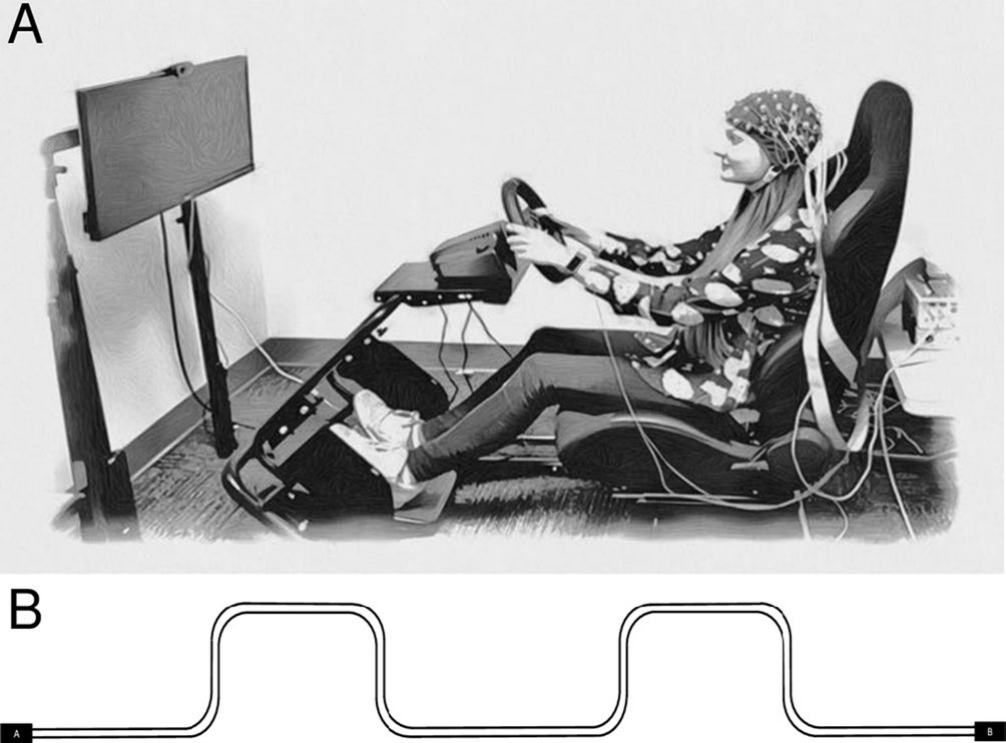
Experimental set-up on a pseudoparticipant (**A**), and the driving course (**B**). The start and end of the driving course was counterbalanced to ensure the direction of the first chicane.

Stimulus presentation and behavioural data collection was controlled via a PC running Drivesim 6 software (York Driving Technologies, CA). Logitech (Driving Force G920) wheel and pedals were used to perform the driving task. A custom-built simulator frame (Dominator series 2, Hyperdrive Australia Ltd.) was used in the current study. Participants were seated approximately 120 cm from the centre of the screen (29-inch monitor) whilst performing the driving task in the driving simulator (Fig. 1, Panel A).

The driving course was set-up as an approximately 60-min drive at 50 km/h through a driving scene with no other vehicles, buildings or trees in the landscape to maximise the monotonous nature of the environment. The total duration of the drive slightly differed between participants depending on their speed throughout the drive; if a speed of 50 km/h was consistent throughout the drive, the total drive duration would be 60 min. This was to ensure that participants had the opportunity to view all the presented peripheral stimuli embedded within the driving course. The route involved driving along a straight road for the majority of the drive duration with the exception of eight chicanes. Two chicanes (one left and one right) were designed to occur at approximately 15-min intervals. The driving scene consisted of a blue surface on the top of the screen (i.e., representing the sky), a grey road and a green untextured surface (i.e., representing grass) outside the road. Participants could view their driving speed and the bonnet of the car whilst driving. Road signs indicating a chicane ahead and speed signs displaying 50 km/h were placed throughout the drive. Participants were given an equal number of left- and right-sided chicanes throughout the drive (see Fig. 1, Panel B). Whilst driving, 96 circular grey stimuli, appearing sequentially at varying eccentricities to the left and right of the screen, were presented. Stimulus presentation was for 1500 ms and the response window was set to a maximum of 2000 ms following stimulus onset. Stimuli were presented in a pseudo-randomised order with each half of the drive containing 48 stimuli. Within each half, there were eight repetitions of the six basic factorial combinations deriving from two sides (left and right) and three different horizontal eccentricity locations (2°, 4°, 6° from the centre of the screen). All stimuli were presented along the vertical midpoint of the screen. Stimuli were 0.47° in visual angular diameter. Participants responded to stimuli using levers attached to the steering wheel.

The following data was used for analysis: reaction time following correctly identified stimulus presentations, the number of omissions (i.e., the trials that participants did not respond to stimuli), lane variability (calculated as the standard deviation of the road position) and time within 10% of the speed limit (calculated as the total time in minutes spent within the speeds 45–55 km/h). The drive duration was split into two halves for analysis.

#### Karolinska Sleepiness Scale (KSS)

A 9-point rating scale was used to assess subjective sleepiness prior to and following the driving task. The Karolinska sleepiness scale ranges from 1 “*extremely alert*” to 9 “*very sleepy*”^35^. The KSS is a widely used measure of subjective sleepiness and has demonstrated validity for assessing alertness^36^.

### Physiological data acquisition

#### Electroencephalography (EEG) recording and data processing

EEG was recorded using 64 active EEG electrodes using the Biosemi Active two system (Biosemi, Netherlands). Recordings were grounded using common mode sense and driven right leg electrodes (http://www.biosemi.com/faq/cms&drl.htm). Prior to testing commencement, electrode offset was minimised and stabilised. The EEG was recorded at 2048 Hz and downsampled to 512 Hz. The data was cleaned with a high pass of 1 Hz and bad channels were removed based on a Kurtosis threshold greater than 5. Bad components were removed using the ICLabel plugin in EEGlab^37^ with a signal artefact threshold of .8 being removed using this method. An independent component analysis (ICA) was run using runica with default settings. Removed channels were interpolated using the spherical spline method. Data were then low pass filtered at 20 Hz and Fast fourier transform (FFT) was conducted using the Pwelch method^38^ with a window length of 2048. Electrode AFz was used as the reference electrode. FFT was used to extract frequency bands corresponding to alpha activity. The commonly used frequency range of 8–12 Hz for alpha was examined in the current study^39,40^. The power was normalised to the average power per channel. EEG processing was performed using the EEGLAB toolbox (Version 14.1.2b)^41^ in MATLAB (Version 2019b; Mathworks, USA).

EEG alpha relative power for each participant was obtained and temporally split into two halves based on each participant’s total drive duration on the driving simulator for statistical analyses. The brain regions were further separated into the left and right hemispheres and separated into frontal and parietal-occipital regions as these areas commonly generate alpha^42,43^. The following EEG regions were examined for analysis: P4, P6, P8, PO4, PO8, O2 (right hemisphere parieto-occipital), F2, F4, F6, F8 (right hemisphere frontal), P3, P5, P7, PO3, PO7, O1 (left hemisphere parieto-occipital), and F1, F3, F5, F7 (left hemisphere frontal). Alpha relative power within these EEG regions were collapsed to obtain an average for each region (i.e., the average value of F1, F3, F5, & F7 formed the left frontal region average relative power value).

#### Galvanic skin response (GSR) recording and data processing

Galvanic skin response embedded into the EEG system was used for skin conductance data recording (Biosemi, Netherlands). Two GSR electrodes were placed on the lower portion of the palm on the non-dominant hand (as determined by the FLANDERS)^34^. A conductive gel was applied between the electrodes and the skin to improve sensors/skin contact. To obtain good skin–electrode contact, tape was used to keep electrodes in place. GSR was recorded at 2048 Hz. The measured GSR frequency ranged between .01 and 5 Hz, and the calculated sympathetic nervous system (SNS) activity was between .05 and .24 Hz^44^. Skin conductance is widely used to measure autonomic nervous system activity^45^ and is a sensitive measure of vigilance^46^. In the current study, normalised SNS range (i.e., .01 to 2 Hz) value was used as the skin conductance measure and the change in power over time was used in the analysis. Similar to the EEG analysis, GSR activity was split into two halves relative to the duration of drive. The room temperature was maintained at approximately 22 ± 2 °C for all participants.

### Procedure

The study was conducted at the Cognitive Ageing and Impairment Neurosciences Laboratory (CAIN), located at the University of South Australia Magill Campus. Participants attended the laboratory for testing and provided written informed consent prior to participation. Following consent, participants completed a series of questionnaires which measured handedness and a pre-drive subjective sleepiness scale (i.e., Karolinska Sleepiness Scale [KSS]). Participants were then set up with an EEG cap and GSR electrodes before completing a driving task in a simulator for approximately 1 h. Refer to Fig. 1 (Panel A) for the experimental set-up on a pseudoparticipant who provided informed consent to publish the identifying image. EEG and GSR were recorded throughout the drive.

The height of the driving simulator screen was adjusted for each participant to ensure the centre of the screen was approximately at eye level and participants could see the whole screen clearly. Participants were unaware of the course they were driving through (depicted in Fig. 1, Panel B). Half the participants started the course at location A and the other half started at B to ensure that the direction of the first chicane was counterbalanced. Participants were given an opportunity to familiarise themselves with the task by completing a practice drive that lasted approximately 4 min. If the participant was unable to complete the practice drive successfully (i.e., they struggled to maintain the speed limit or were unable to detect stimuli), they were given another opportunity to complete the practice drive (only one participant completed the practice drive twice). Participants were instructed to maintain a speed limit of 50 km/h and remain in the middle of the three-lane road. Whilst driving, participants were also instructed to respond to stimuli appearing to the left and right side of the screen, pressing a left button when a left-sided stimulus appeared and the right button when a right-sided stimulus appeared. The levers were attached to the steering wheel. Participants were not told to prioritise either task.

A webcam was used to monitor whether participants were adhering to task instructions. If participants were speeding excessively (i.e., more than 20 km/h above the speed limit for a duration longer than 2 min, they were asked to adhere to the speed limit). Participants were informed that the total duration of the task was approximately 1 h. Upon completion of the driving task, participants filled out more questionnaires (e.g., post-drive KSS). Total testing time lasted approximately 3 h. All procedures were in accordance with the National Statement on Ethical Conduct in Human Research.

### Statistical analyses

#### Data analysis

Data were analysed using jamovi version 1.1.9.0^47^ and R^48^. First, data were screened for outliers. Outliers were defined as more than three standard deviations above or below the group mean for all measures. One participant was identified as an outlier (more than 3 SDs below the group mean for behavioural performance) and excluded (see “Participant” section above). On the driving simulator task, none of the participants responded within 100 ms of stimulus presentation, therefore anticipatory responses did not need to be removed.

A series of mixed effects models were conducted to investigate changes in alertness with time-on-task and to investigate the relationship between spatial attention and alertness. Participant ID was a random effect in all mixed-effects models. Post hoc comparisons with Holm corrections were used to account for multiple comparisons within models. No corrections for multiple comparisons between models were applied. Partial eta^2^ effect sizes were calculated independently from the mixed effects modelling.

#### Changes in indices of alertness (KSS, EEG and GSR) with time-on-task

A series of mixed effect models were conducted to examine alertness changes with time-on-task. A mixed effects model with an outcome variable of KSS rating and predictors of time (pre-drive/post-drive) and handedness (left-handed/right-handed) was used to determine changes in subjective sleepiness from pre-drive to post-drive. Two mixed effects models with alpha relative power and normalised SNS power as outcomes and a predictor of time (i.e., split by first half and second half of drive) were conducted. Handedness was a predictor in all models, and hemisphere (left/right) and lobe (Parietal/Occipital) were further predictors in the alpha model.

#### Changes in driving simulator performance with time-on-task

Two separate mixed effects models with outcome variables lane deviation and time spent within 10% of the speed limit, with a predictor of time (first half/second half), handedness (left-handed/right-handed) and an interaction of time by handedness were conducted.

#### Changes in spatial attention with time-on-task

Two mixed more effects models with reaction time to peripheral stimuli, and omission of stimuli as outcome variables and time (first half/second half), location (left 6°, left 4°, left 2°, right 2°, right 4°, and right 6°) and handedness (left/right) as predictors were also conducted. Reaction time to peripheral stimuli and omissions of stimuli (i.e., the number of omitted responses to peripheral stimuli) were outcome variables in separate models. Another two separate mixed effects models, one for left-handers and one for right-handers were conducted. Each of these stratified models contained fixed factors of time and location, and reaction time as the outcome variable. Each of the two models contained fixed factors of time and location, and reaction time as the outcome variable.

#### Relationship between alertness and spatial attention measures

A series of exploratory Spearman’s rho correlations assessed whether changes in physiological variables (GSR and alpha) correlated with changes in spatial performance (i.e., reaction time). Change scores were computed for GSR, alpha, and reaction time to peripheral stimuli at each location identified as statistically significant, by subtracting the first half of the drive from the second half of drive. No corrections for multiple comparisons between models were applied.

## Results

### Changes in indices of alertness (KSS, EEG and GSR) with time-on-task

**Figure 2:**
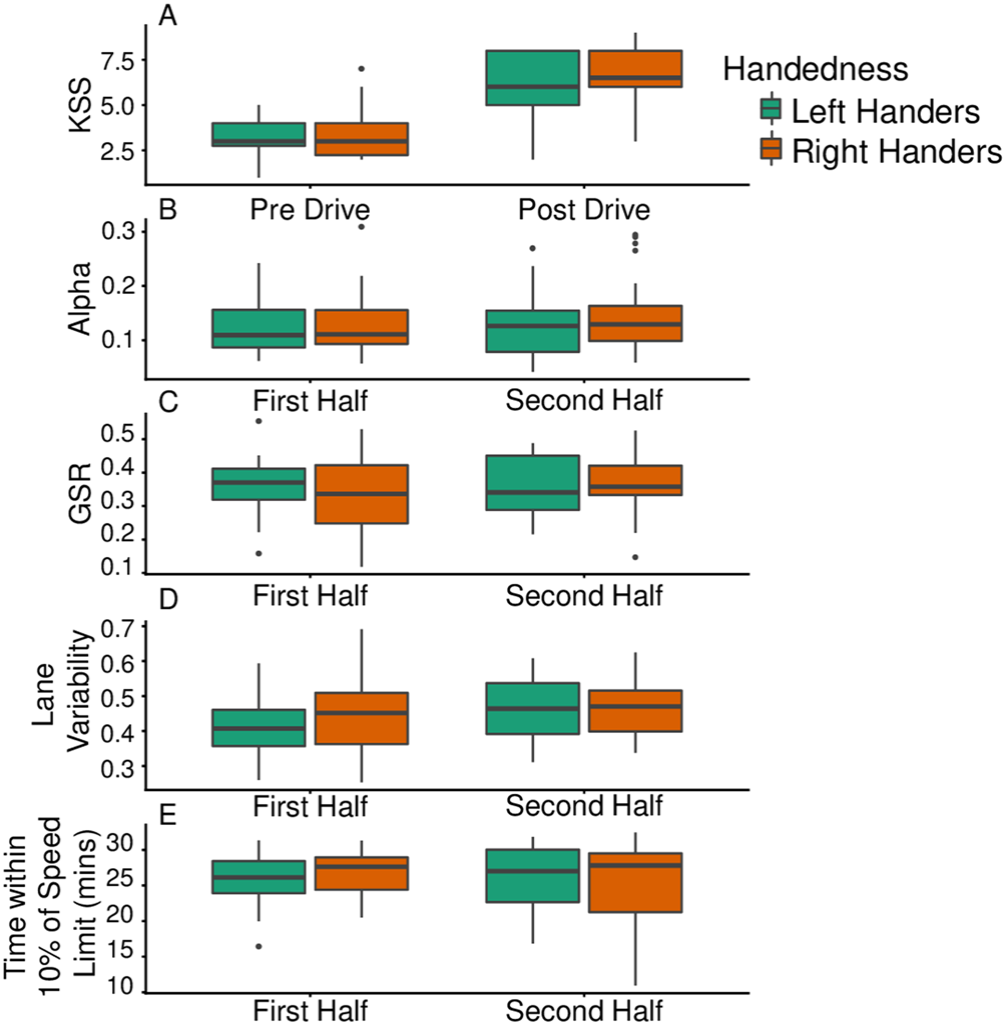
Changes in KSS (**A**), alpha (**B**), GSR (**C**), lane variability (**D**) and time spent within 10% of the speed limit (**E**), separated by handedness.

A main effect of subjective sleepiness (as indexed by the KSS) revealed increased self-reported sleepiness post-drive compared to pre-drive (*p* < .001) in a mixed model. See Fig. 2. There was a main effect of time for alpha (*p* = .005) with alpha relative power increasing from the first half of the drive compared to the last. There was no main effect of time on GSR (*p* = .387). There was no main effect of handedness or a time by handedness interaction in any of the three models. Refer to Table 1 for F values, degrees of freedom, *p* values and Holm-corrected post hoc comparisons relative to time of testing, for each measure derived from the mixed effects models. The calculated partial eta^2^ effect size was large for KSS (.75), moderate for alpha (.14), and small for GSR (.02).

**Table 1:** F values, degrees of freedom, *p* values and holm corrected post hoc comparisons with corrections relative to time-on-task and handedness, for each KSS, Alpha and GSR from the mixed effects models.

Outcome variable	Predictor	F	df	*p*	Significant post hoc comparisons
KSS	Time (pre-drive and post-drive)	120.7	1, 40	< .001	Pre-drive < post-drive
	Handedness	2.67	1, 40	.110	
	Time * handedness	.866	1, 40	.358	
Alpha	Time	7.86	1, 283	.005	1st < 2nd
	Hemisphere	1.98	1, 283	.161	
	Lobe	178.5	1, 283	< .001	Frontal < parieto-occipital
	Handedness	.504	1, 40	.482	
	Time * hemisphere	.0009	1, 283	.977	
	Time * lobe	.915	1, 283	.340	
	Time * handedness	.453	1, 283	.501	
	Hemisphere * handedness	.205	1, 283	.651	
	Hemisphere * lobe	3.94	1, 283	.048	Frontal left < frontal right, parieto-occipital left, parieto-occipital right; parieto-occipital left > frontal right
	Handedness * lobe	.627	1, 283	.294	
	Time * hemisphere * handedness	.003	1, 283	.956	
	Time * lobe * handedness	.646	1, 283	.422	
GSR	Time	.764	1, 40	.387	
	Handedness	.072	1, 40	.789	
	Time * handedness	1.45	1, 40	.235	

Hemisphere and lobe are additional predictors in the alpha model.

LH = left-handers, RH = right-handers. Pre-D = pre-drive, post-D = post drive. For post hoc analyses < and > indicate the direction of the effect (e.g., 1st < 2nd refers lower levels of the outcome variable in the first half compared to second half of drive). Only relevant post hoc comparisons reaching statistical significance (*p* < .05) following corrections for multiple comparisons are reported for interactions.

### Changes in driving simulator performance with time-on-task

Lane variability increased with time-on-task (*p* = .008). There was no main effect of time for time spent within 10% of the speed limit. There was no main effect of handedness or an interaction of time by handedness in the two models. The calculated partial eta^2^ effect size was moderate for lane variability (.15), and small for time spent within 10% of the speed limit (.01). See Fig. 2 and refer to Table 2 for post hoc comparisons.

**Table 2:** F values, degrees of freedom, *p* values and holm corrected post hoc comparisons with corrections relative to time-on-task and handedness, for driving simulator variables derived from the mixed effects models.

Outcome variable	Predictor	F	df	*p*	Significant post hoc comparisons
Lane variability	Time	7.81	1, 40	.008	1st < 2nd
	Handedness	.218	1, 40	.643	
	Time * handedness	1.40	1, 40	.244	
Time in safe zone	Time	.481	1, 40	.492	
	Handedness	.047	1, 40	.829	
	Time * handedness	1.31	1, 40	.260	
Reaction time to stimuli	Time	10.2	1, 3397.6	.001	1st > 2nd
	Location	130.9	5, 3399.4	< .001	L1 > L2, L3, L4, L5; L3 < L2, L5; L4 < L2, L5; L6 > L1, L2, L4, L5
	Handedness	2.15	1, 39.8	.150	
	Time * location	.720	5, 3397.4	.609	
	Time * handedness	4.31	1, 3397.6	.038	1st RH < 2nd RH
	Location * handedness	.714	5, 3399.4	.613	
	Time * location * handedness	3.49	5, 3397.4	.004	
Omissions	Time	6.19	1, 440	.013	
	Location	27.36	5, 440	< .001	L1 > L2, L3, L4, L5; L6 > L2, L3, L4, L5
	Handedness	.23	1, 40	.632	
	Time * location	2.41	5, 440	.049	1st L6 > 1st L2, 1st L3, 1st L4, 1st L5; 2nd L6 > L2, 2nd L3, 2nd L4, 2nd L5; 1st L1 > 1st L3, 1st L4, 1st L5; 2nd L1 > 2nd L2, 2nd L4, 2nd L5
	Time * handedness	.05	1, 440	.816	
	Location * handedness	7.51	5, 440	< .001	RH L6 > RH L3, RH L4, RH L5, LH L6; LH L2 < LH L6
	Time * location * handedness	.97	5, 440	.432	

Location is an additional predictor in the reaction time and omissions to peripheral stimuli models.

LH = left-handers, RH = right-handers. For post hoc analyses < and > indicate the direction of the effect (e.g., LH 1st < LH 2nd refers to left-handers’ performance in the first half of the drive is lower than that of left-handers’ performance in the second half of drive). L1 = location 1, L2, location 2, L3 = location 3, L4 = location 4, L5 = location 5, L6 = location 6. Only relevant post hoc comparisons reaching statistical significance (*p* < .05) following corrections for multiple comparisons are reported for interactions.

### Changes in spatial attention with time-on-task

**Figure 3:**
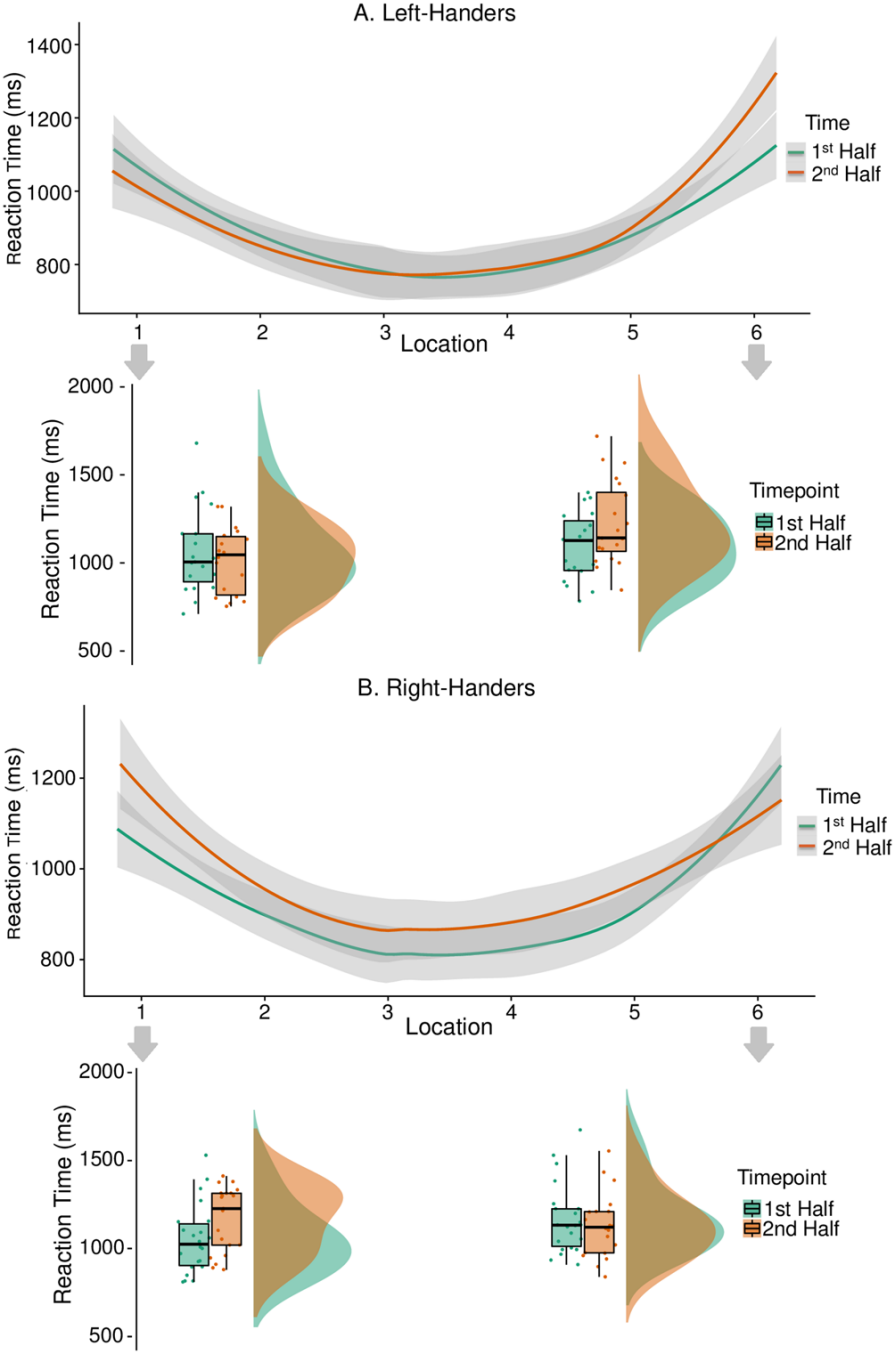
Interaction between time-on-task, stimulus location and handedness. Differences as a function of handedness and time-on-task become particularly evident in performance to the most lateral stimulus locations. (**A**) Reaction time pattern of left-handers with the detailing of performance at locations 1 & 6. (**B**) Reaction time pattern of right-handers with the detailing of performance at locations 1 & 6. Right-handers show increased reaction time to the leftmost periphery (location 1) with time-on-task. Shaded area represents 95% confidence intervals.

Reaction time to peripheral stimuli increased with time-on-task (*p* < .001). A location by time by handedness interaction reached statistical significance (*p* = .004). See Table 2. The calculated partial eta^2^ effect size for reaction time was large (.30). The mixed models stratifying left-handers and right-handers revealed a statistically significant location by time interaction for right-handers (*p* = .023), but not left-handers (*p* = .146). Refer to Fig. 3 for the interaction pattern for both left-handers (Panel A) and right-handers (Panel B). See Table 3 for mixed models F values, degrees of freedom, *p* values, and Holm corrected *p* values.

**Table 3:** F values, degrees of freedom, *p* values and holm corrected post hoc comparisons with corrections relative to time-on-task and location separated by handedness for models with the outcome reaction time.

Handedness	Predictor	F	df	*p*	Significant post hoc comparisons
1. Left-Handers	Time	.621	1, 1630	.431	
	Location	68.97	5, 1631	< .001	L2 > L3, L4; L6 > L1, L2, L3, L4, L5; L1 > L2, L3, L4, L5; L5 > L3, L4
	Time * location	1.643	5, 1630	.146	
2. Right-Handers	Time	14.01	1, 1767	< .001	1st half < 2nd half
	Location	64.23	5, 1768	< .001	L1 > L2, L3, L4, L5; L2 > L3, L4; L5 > L3, L4; L6 > L2, L3, L4, L5
	Time * location	2.62	5, 1767	.023	1st half L1 < 2nd half L1; 1st half L1 > 1st half L2, 1st half L3, 1st half L4, 1st half L5; 1st half L6 > 1st half L2, 1st half L2; 1st half L6 > 1st half L3, 1st half L4, 1st half L5; 2nd half L6 > 2nd half L2, 2nd half L3, 2nd half L4, 2nd half L5; 2nd half L1 > 2nd half L2, 2nd half L3, 2nd half L4, 2nd half L5

For post hoc analyses < and > indicate the direction of the effect. For example, L2 > L3 reflects higher reaction time at location 2 compared to location 3. Only relevant post hoc comparisons reaching statistical significance (*p* < .05) following corrections for multiple comparisons are reported for interactions.

**Figure 4:**
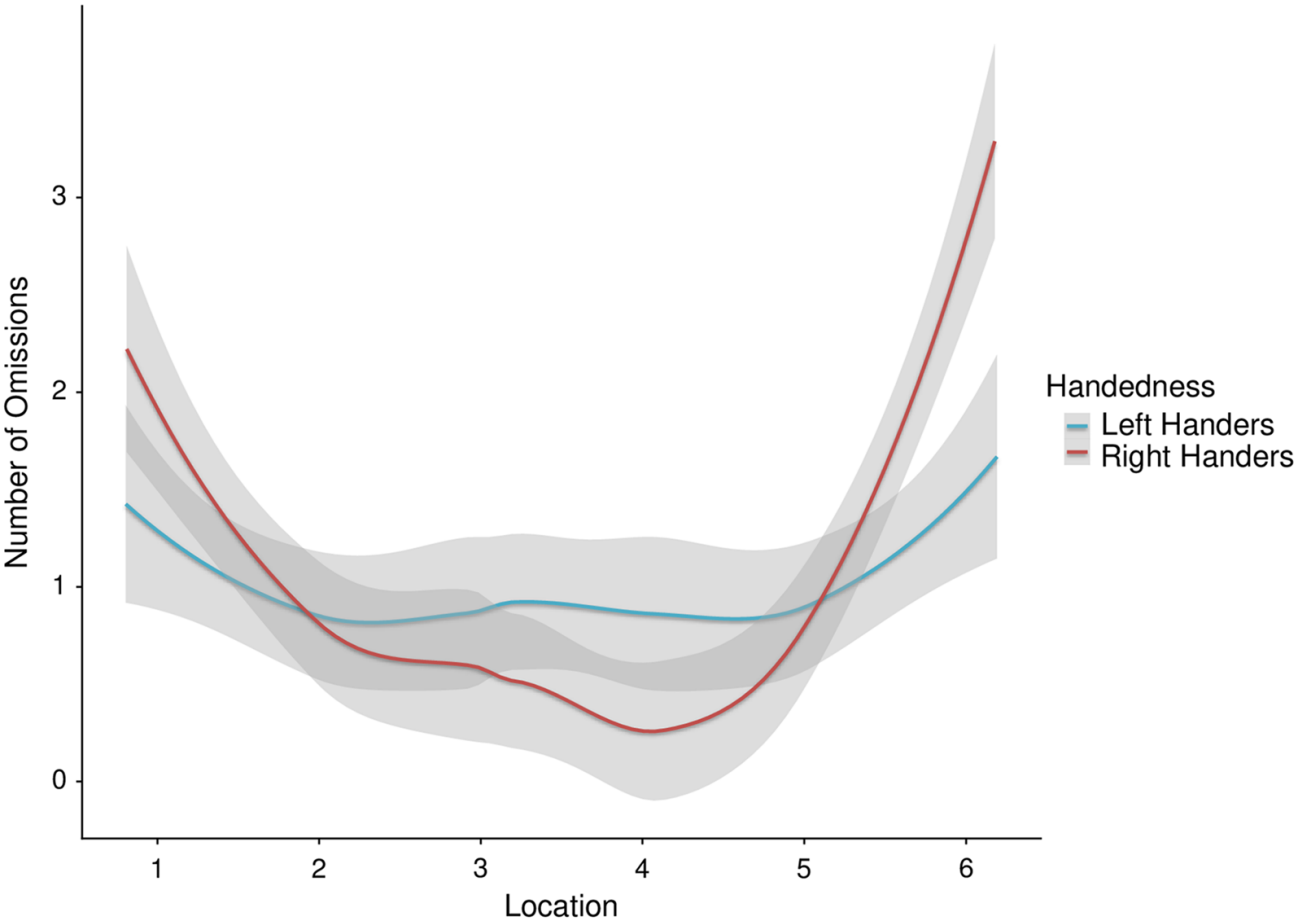
A handedness by location interaction revealed right handers show an increased number of omissions in the rightmost periphery compared to left handers. Smoothed means and shaded area representing 95% confidence intervals are displayed.

Peripheral stimuli omissions increased with time-on-task (*p* = .013). A handedness by location interaction reached statistical significance (*p* < .001) showing right-handers omitted more stimuli in the rightmost periphery compared to left-handers (Fig. 4). There was no time by handedness interaction for omissions. Refer to Table 2 for post hoc comparisons from the mixed effects models. The calculated partial eta^2^ effect size for peripheral stimuli omissions was moderate (.16).

### Relationship between alertness and spatial attention measures

**Figure 5:**
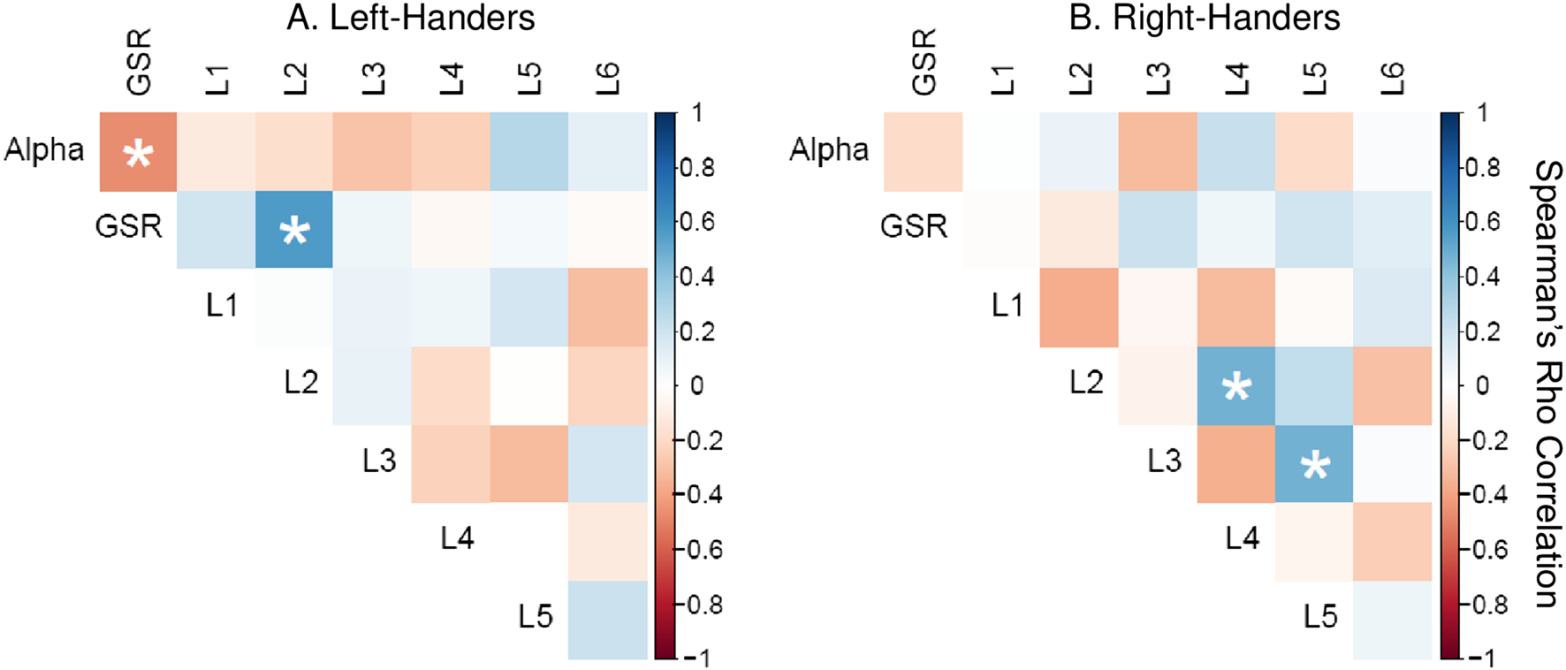
Spearman’s Rho correlations between physiological alertness (GSR and alpha) and reaction time to all six locations for left-handers (**A**) and right-handers (**B**). Correlations reaching significance (*p* < .05) are highlighted. L1 = location 1. Positive values for RT indicate slower RT in the second half than first half of drive (i.e., time-on-task), positive GSR values indicate higher SNS derived alertness with time-on-task, and positive alpha indicates higher alpha power (indicating lower alertness) with time-on-task.

No evidence for reliable relationships between physiological measures (alpha, GSR) and reaction times to stimuli presented at the six spatial locations were observed. There was one exception, however, between GSR and reaction time to stimuli at location 2 (i.e., the second leftmost peripheral stimulus location) in left-handers, with a moderate effect size. See Fig. 5 for correlations.

## Discussion

The current study aimed to investigate the relationship between spatial attention and alertness under prolonged simulated driving in left-handers and right-handers. More specifically, we aimed to uncover whether there are shifts in spatial attention during simulated driving, and whether handedness influences the spatial attention and alertness relationship with time-on-task.

### Changes in indices of alertness and driving performance with time-on-task

We observed the expected decrease in alertness as indicated by an increase in subjective sleepiness (measured via KSS) and an increase in alpha relative power with time-on-task. These findings are consistent with previous literature examining time-on-task effects on subjective sleepiness and alpha^19,49,50^. Another assay of alertness employed in the current study was sympathetic nervous system (SNS) activity as measured by GSR. No SNS activity changes were observed with time-on-task. While unexpected, our finding aligns with a previous study which also found no changes in GSR with time-on-task whilst performing an alertness decreasing task, which lasted 2 h^51^. A plausible reason for failing to detect changes in SNS activity might be the result of a flooring effect due to the sedentary driving task employed. While there were a lack of changes in SNS activity, the other two measures clearly indicate decreases in alertness. The subjective sleepiness and alpha measures of alertness were not modulated by handedness, revealing that both left-handers and right-handers decreased in alertness with time-on-task.

Decreases in alertness indexed by self-reports and alpha power were mirrored by decreases in driving performance. An increase in lane variability with prolonged task exposure was identified in the current study. This finding is consistent with previous studies using driving simulators^2,52^. We found no statistically significant differences in speed with time-on-task, demonstrating our participants did not deviate from a speed within 45–55 km in both the first and second half of the drive. This might be indicative of the relatively low speed limit (i.e. 50 km) participants adhered to, being easier to maintain than higher speeds, and the nature of the task not requiring for speed changes throughout the drive. However, performance decrements were also found in the detection of stimuli presented within the driving task, where we observed increases in reaction time and omissions in the second half compared to the first half of the drive. Again, these effects are typically found in detection tasks with lowered alertness^2,16,17^. Similar to our assays of alertness, measures of driving performance (lane variability and time spent within 10% of the speed limit) were not modulated by handedness. Finally, the pattern of the reaction times across the six location shows the typical U-shape with slower and faster reactions times in the periphery and centre, respectively^17,53,54^. Having established that the prolonged driving task elicited the expected decreases in alertness, driving performance and slower reaction times, we now turn the discussion to the effects of the prolonged driving task on spatial attention as a function of handedness.

### Spatial attention as a function of time-on-task and handedness

There was clear evidence that prolonged driving affects left- and right-handers’ ability to react to stimuli presented in the environment differently. These differences become particularly evident in the detection performance to the most lateral targets used in this study (see Fig. 3). Right-handers became significantly slower to react to the leftmost peripheral stimulus location in the second compared to first half of the drive. This finding is in line with previous studies demonstrating a rightward shift in attention with time-on-task and under lowered alertness conditions^19,55–57^. Left-handers seem to show the opposite reaction time pattern of right-handers: with slower reaction times to right-sided stimuli in the second compared to the first half of the drive. However, the differences at the rightmost lateral stimulus were statistically not significant for left-handers when the analyses were corrected for multiple comparisons (*p* uncorrected = .017).

A three-way interaction of time by location by handedness did not reach significance for the number of omissions to stimuli, suggesting that there were no large differences in omissions of stimuli with time-on-task relative to the stimulus location as a function of handedness. A reason for this null-finding could be that reaction time might be a more sensitive measure than omissions in detecting subtle differences in performance. Our findings revealing more omissions in the rightmost periphery in right-handers might also reflect pseudoneglect. This is in line with a study comparing spatial bias on the Landmark task and a driving task revealing a leftward bias in both tasks, indicative of the pseudoneglect phenomenon^58^. There was no clear evidence for a tunnelling effect as time progressed. It is important to note that the location specific analyses were based on a relatively small number of stimuli (e.g., eight stimuli within each location at each half of the drive), and thus some comparisons may lack appropriate statistical power. The relatively small number of stimuli was chosen in order to reduce the possibility of an alerting effect being introduced due to frequent stimulus presentation.

Overall, our handedness findings revealing differences in reaction times to stimuli are in line with findings by a study who showed left-handers and right-handers to differ in their spatial attention and alertness relationship^32^.

### Relationships between physiological indices of alertness and spatial attention

Correlations examining the relationship between alertness, measured via alpha and GSR, and spatial bias, measured via responses to target stimuli, failed to confirm a reliable relationship between changes in these measures (see Fig. 5). This finding was unexpected as we predicted that changes in physiological alertness measures would associate with changes in spatial attention^19^. An explanation for the lack of associations could be the relatively crude physiological measures employed in the current study. While we looked for changes in average alpha power across the two halves of the drive, investigating changes in alpha immediately before target onset might have been more revealing. For example, a study found changes in pre-target alpha over the right relative to the left hemisphere were associated with a rightward shift in spatial biases^19^.

We found evidence for differences in alpha across different brain regions (e.g., higher alpha in parieto-occipital lobe than frontal lobe and higher alpha in the right compared to left frontal lobe, see Table 1), but these differences across regions were not modulated by time or handedness. Thus, the EEG measures in our study did not provide insights into the interactions between alertness and spatial attention. It cannot be ruled out that our sample of 22 right-handers and 20 left-handers was underpowered to detect small interaction effects. Importantly, however, the EEG measures did confirm that the time-on-task manipulation decreased alertness with time as indexed by an increase in alpha power as has been previously shown^43,49^ and demonstrated a moderate effect size.

### Implications of study findings

Although the EEG findings were inconclusive regarding the mechanisms underlying the interactions between alertness and spatial attention, the differences between left- and right-handers in this study suggest that the dominant model for the interactions proposed by Corbetta and Shulman^24^ may need some refinement. The direction of the attentional shifts proposed by the model (to the left and right with high and low levels of alertness, respectively) might not necessarily hold true for left-handers. The reason for not showing the same pattern could be related to differences in the lateralisation of the DAN and VAN between left- and right-handers^30,31^ which may consequently influence the spatial attention and alertness relationship differently in these two groups. Hence, it is possible that anatomical differences related to the location and connectivity of the DAN and VAN in left- and right-handers could be influencing the direction of attentional shift. However, there is need for further investigation into the localisation of the DAN and VAN to understand the spatial attention and alertness relationship better.

Research on the relationship between alertness and spatial attention has been largely contained to tightly controlled laboratory studies employing paper and pencil or computerised tasks^20^. In the current study, we attempted to increase real-world applicability of computerised spatial bias tasks by assessing the interactions between alertness and spatial attention while observing participants’ driving behaviours in a safe and controlled environment. Although still based on an artificial task, the current findings confirm the possibility that these interactions could have safety implications for activities such as driving. For example, the right-handers’ slowed responses and omission of left-sided compared to right-sided peripheral stimuli could potentially translate to delays in detecting relevant left-sided traffic information and dangers in the real-world. To what degree the stimuli used in this study are reflective of potential real-world dangers (e.g., a pedestrian walking onto the street) is unknown given inconsistencies in real-world applicability of driving simulators^59^. However, given the high reliance on driving in today’s society, and the increasing number of hours spent commuting^60^, the potential implications resulting from impaired responding to the environment can be significant, on both an individual and economic level. Hence, it is critical to further investigate the factors that influence the ability to respond to the environment. Future research can benefit from investigating whether the effect of asymmetries in attending to the periphery is present in differing road conditions. Here, the simulated driving was conducted in a monotonous environment to decrease alertness, but busy environments and factors such as light traffic may alert drivers and help counteract time-on-task effects^3^.

## Conclusion

Our study examined the effect of time-on-task on spatial bias under simulated driving performance. We found evidence of attentional shifts as a function of handedness as time-on-task progressed. Right-handers reacted slowly to the leftmost targets, while left-handers showed the opposite pattern (although not statistically significant) in the second compared to first half of the drive. As the validity of driving simulators in predicting on-road driving is currently ambiguous, the real-world implications of our findings remain unclear. However, given the potential safety implications for real-world driving inattention, further investigations into the transfer effects of these findings are required.
